# Hyperglycemia and Diabetes Downregulate the Functional Expression of TRPV4 Channels in Retinal Microvascular Endothelium

**DOI:** 10.1371/journal.pone.0128359

**Published:** 2015-06-05

**Authors:** Kevin Monaghan, Jennifer McNaughten, Mary K. McGahon, Catriona Kelly, Daniel Kyle, Phaik Har Yong, J. Graham McGeown, Tim M. Curtis

**Affiliations:** Centre for Experimental Medicine, Queen’s University of Belfast; Indiana University School of Medicine, UNITED STATES

## Abstract

Retinal endothelial cell dysfunction is believed to play a key role in the etiology and pathogenesis of diabetic retinopathy. Numerous studies have shown that TRPV4 channels are critically involved in maintaining normal endothelial cell function. In the current paper, we demonstrate that TRPV4 is functionally expressed in the endothelium of the retinal microcirculation and that both channel expression and activity is downregulated by hyperglycaemia. Quantitative PCR and immunostaining demonstrated molecular expression of TRPV4 in cultured bovine retinal microvascular endothelial cells (RMECs). Functional TRPV4 activity was assessed in cultured RMECs from endothelial Ca2+-responses recorded using fura-2 microfluorimetry and electrophysiological recordings of membrane currents. The TRPV4 agonist 4α-phorbol 12,13-didecanoate (4-αPDD) increased [Ca^2+^]_i_ in RMECs and this response was largely abolished using siRNA targeted against TRPV4. These Ca^2+^-signals were completely inhibited by removal of extracellular Ca^2+^, confirming their dependence on influx of extracellular Ca^2+^. The 4-αPDD Ca^2+^-response recorded in the presence of cyclopiazonic acid (CPA), which depletes the intracellular stores preventing any signal amplification through store release, was used as a measure of Ca^2+^-influx across the cell membrane. This response was blocked by HC067047, a TRPV4 antagonist. Under voltage clamp conditions, the TRPV4 agonist GSK1016790A stimulated a membrane current, which was again inhibited by HC067047. Following incubation with 25mM D-glucose TRPV4 expression was reduced in comparison with RMECs cultured under control conditions, as were 4αPDD-induced Ca^2+^-responses in the presence of CPA and ion currents evoked by GSK1016790A. Molecular expression of TRPV4 in the retinal vascular endothelium of 3 months’ streptozotocin-induced diabetic rats was also reduced in comparison with that in age-matched controls. We conclude that hyperglycaemia and diabetes reduce the molecular and functional expression of TRPV4 channels in retinal microvascular endothelial cells. These changes may contribute to diabetes induced endothelial dysfunction and retinopathy.

## Introduction

Retinopathy is a sight threatening complication of diabetes and a major cause of vision loss[1,2]. Its pathology primarily affects the retinal microcirculation and current treatments target late stages of the condition through laser ablation or the use of anti-VEGF drugs[3–5]. Good control of blood glucose and blood pressure remain central to delaying the onset and slowing the progress of diabetic retinopathy[6]. However, better understanding of its pathogenesis may allow molecular targets to be identified for novel, retinopathy specific treatments. Reduced autoregulatory changes in vascular diameter in responses to changes in O_2_, vascular transmural pressure and retinal stimulation with a flickering light are among the earliest defects of retinovascular function seen in diabetes[7–13]. This suggests that molecules believed to play a role in the control of vascular tone may be implicated in the diabetic changes in the eye.

The fourth member of the vanilloid sub-family of transient receptor potential channels (TRPV4) suggests itself as one such molecule. It is a Ca^2+^-permeable non-selective cation channel that can be activated by range of stimuli[14]. The channel is expressed in both vascular endothelium and smooth muscle, and plays an important role in vasodilator responses. Endothelial TRPV4 has been shown to be activated by mechanical stimuli such as shear stress and reduced intravascular pressure[15–19], agonist binding to Gq coupled receptors [20,21] and directly by cytochrome P450 (CYP) epoxygenase-derived epoxyeicosatrienoic acids (EETs)[19,22]. The resulting rise in endothelial [Ca^2+^] leads to relaxation of the adjacent smooth muscle and vasodilatation by a range of mechanisms including activation of small and intermediate conductance Ca^2+^-sensitive K^+^-channels in the endothelium and signaling via both the endothelial derived hyperpolarizing factor (EDHF) and nitric oxide (NO) pathways[15,21,23,24]. Activation of TRPV4 in vascular smooth muscle may augment endothelial dilator mechanisms through a TRPV4-ryanodine receptor-large conductance Ca^2+^-sensitive K^+^-channel (BK) mechanism[23,25]. TRPV4 channels have also been implicated in the control of capillary permeability in the lungs[26] and in stimulating angiogenic processes in endothelial cells from human brain capillaries and breast carcinoma tissue[27,28]. It seems likely, therefore, that disease-induced changes in TRPV4 expression could have important pathophysiological consequences.

In the current study we have demonstrated that TRPV4 is expressed in cultured endothelial cells of the retinal microcirculation and have validated protocols allowing functional expression of TRPV4 in the cell membrane to be quantified. TRPV4 expression was downregulated at both molecular and functional levels after only 72 hours exposure of retinal microvascular endothelial cells to high glucose in culture. Furthermore, we show that the molecular and protein expression of endothelial TRPV4 channels is decreased in the retinal microvasculature after 3-months’ experimental diabetes in rats. These data suggest that loss of TRPV4 function may play a role in the early disruption of retinovascular function and merits further investigation as a potential therapeutic target in diabetic retinopathy.

## Methods

### Primary Cell Culture

Primary retinal microvascular endothelial cells (RMECs) were prepared from bovine eyes as previously described[29]. Cells from a minimum of 3 different extractions were used between passages 2 and 5 in all protocols, and were treated for 72hr with DMEM containing one of the following: 5mM D-glucose (euglycaemic control); 25mM D-glucose (hyperglycaemia); 5mM D-glucose + 20mM mannitol or 5mM D-glucose + 20 mM L-glucose (osmotic controls). Ca^2+^ microfluorimetry and electrophysiological recordings were made in the presence of the same glucose and/or mannitol concentrations as was present in the culture medium for the relevant RMECs.

### siRNA Transfections

Cells were transiently transfected with 30 nM small interfering RNA (siRNA) for 48 hr using TurboFect transfection reagent (Fermentas Life Sciences, Ontario, Canada). Cells were treated with siRNA for bovine TRPV4 (Qiagen Crawley, UK; based on the sequence 5’AGCCCCACATCGTCAACTAC3’) or negative control siRNA (Qiagen; based on the sequence 5’AATTCTCCGAACGTGTCACGT3’). Following transfection, cells were washed and cultured for a further 72hr prior to experimentation. Experiments were conducted over several independent passages and gene silencing confirmed by quantitative PCR and Western Blot.

### Streptozotocin diabetic rat model

All animal procedures were approved by Queen's University of Belfast Animal Welfare and Ethical Review Body (AWERB) and authorized under the UK Animals (Scientific Procedures) Act 1986 (Home Office Project License Number: PPL2654). Male Sprague-Dawley rats (200–250 g) were rendered diabetic by a single intraperitoneal injection of streptozotocin in citrate buffer (STZ; 60 mg/kg)[30]. Animals with glycosylated haemoglobin levels >10% at sacrifice (GHb, Helena Biosciences, UK) were included in the diabetic group. Citrate buffer injected age-matched rats were used as controls. Rats were anesthetized with CO_2_ and killed by cervical dislocation. Retinal arterioles were mechanically isolated in low Ca^2+^ Hanks’ solution and TRPV4 mRNA levels assessed using quantitative RT-PCR. Retinas from separate groups of animals were used for TRPV4 immunolabelling studies.

### Quantitative RT-PCR

Total RNA was extracted from retinal arterioles and RMECs using an RNeasy Micro kit (Qiagen, Crawley, UK)[31]. RNA was quantified using a NanoDrop spectrophotometer (Thermo Scientific, DE, USA) and 200ng of RNA reverse transcribed into cDNA using a Sensiscript Reverse Transcription Kit (Qiagen). Quantitative PCR was performed using a LightCycler rapid thermal cycler system (Roche Diagnostics Ltd, Lewes, UK). Primers (Eurogentech, Southampton, UK) were designed to amplify bovine TRPV4 (forward primer 5’AGCCCCACATCGTCAACTAC3’, reverse primer 5’TGACGAACTTGGTGTTCTCG3’), and rat TRPV4 (forward primer 5’ ACCCTCTGGTCCCCACAAAG3’, reverse primer 5’TGCATGAGCCTCAGCCCTG3’), which were quantified relative to β-actin (bovine, forward primer 5’AGCAAGCAGGAGTACGATGAGT3’, reverse primer, 5’ATCCAACCGACTGCTGTCA3’; rat, forward primer 5’TCCCTGGAGAAGAGCTATGAGCT3’, reverse primer 5’GTTTCATGGATGCCACAGGATT3’) [32].

### Western Blotting

Western blotting was performed as previously described[29]. RMEC protein was precipitated using RIPA buffer and quantified using a bicinchoninic acid (BCA) protein assay kit according to the manufacturer’s protocols (Thermo Scientific Pierce, Rockford, IL, USA). 30 μg of protein was loaded per lane and separated by SDS-PAGE on 8% polyacrylamide gels. Proteins were transferred to a PVDF membrane, which were incubated overnight at 4^°^C with a 1:200 dilution of a rabbit polyclonal antibody against residues 853–871 of rat TRPV4 (Alomone Labs, Jerusalem, Israel; ACC-034). Antibodies targeting β-actin (1:50000, Sigma Aldrich, UK) were used as loading controls. Secondary antibody was applied for 1 hour at room temperature (anti-rabbit 800, 1:10000, LI-COR Biosciences, Cambridge, UK). The blots were imaged with an Odyssey Infrared Imaging System (Li-COR Biosciences).

### Immunolabelling studies

Immunolabelling was carried out on RMECs and rat retinal flatmount preparations. In most experiments, Alomone Labs anti-TRPV4 antibody (Jerusalem, Israel; ACC-034) was used since the specificity of this antibody has previously been confirmed using tissues from TRPV4 knockout mice[33]. RMECs grown on coverslips were fixed with 4% paraformaldehyde for 5 minutes at room temperature and then washed in phosphate buffered saline (PBS) for 1 hour. After blocking and permeabilising (1% donkey serum and 0.05% Triton-X 100 in PBS for 1 hour), cells were incubated overnight with polyclonal anti-TRPV4 antibody (1:200; Alomone Labs, ACC-034) in the absence or presence of relevant blocking peptide (1μg/ml; Alomone Labs). In one set of experiments, a second anti-TRPV4 primary antibody targeting a different epitope of the TRPV4 channel protein was also tested (1:50; Santa Cruz, Dallas, US; SC-98592). Cells were then washed with PBS and secondary antibody applied for 1 hour at room temperature (1:200 donkey anti-rabbit Alexa Fluor488; Life Technologies, Paisley, UK). After washing, cells were incubated with TO-PRO-3 nuclear marker (diluted 1:1000; Life Technologies) for 20 min, and the coverslips mounted on slides in antifade media (Vectashield, Vector Labs Inc, Burlingame, US). For flatmount preparations, rat eyes were were enucleated immediately following sacrifice, the retinae detached and fixed in 4% paraformaldehyde for 20 min and then washed in PBS. Retinae were then placed in permeabilization buffer (0.5% Triton X-100 in PBS), with 5% normal donkey serum (Chemicon International, Temecula, CA, US) to block non-specific antibody binding. They were subsequently incubated for 24 hours with rabbit polyclonal anti-TRPV4 antibody (1:200; Alomone Labs, ACC-034) and in some experiments co-stained with a monoclonal mouse antibody targeting human eNOS (1:200; BD Biosciences, Oxford, UK; 610297) and a goat polyclonal anti-α-smooth muscle actin antibody (1:250; Abcam, Cambridge, UK; Ab21027). For pre-absorption control experiments, the anti-TRPV4 primary antibody was pre-incubated overnight with 1μg/mL blocking peptide (Alomone Labs). After washing, tissues were incubated overnight with donkey anti-rabbit IgG labelled with Alexa-488, donkey anti-mouse IgG labelled with Alexa-568 and donkey anti-goat IgG labelled with Alexa 647 (all 1:200; Life Technologies). Retinae were then washed and mounted in Vectashield. RMECs and flatmount preparations were visualised and images acquired using a Leica TCS SP5 II confocal microscope. TRPV4 immunofluorescence in retinal microvessels from control and diabetic retinas was quantified using Image J (NIH, US), as previously described[31].

### Ca^2+^ fluorescence microfluorimetry

RMECs, plated on 1% gelatin-coated coverslips (zero thickness), were incubated with the membrane permeable Ca^2+^ indicator dye fura 2-AM (5 μM) for 30min at 37°C (AnaSpec, EGT, Liege, Belgium). They were then washed and the coverslips, with the cells attached to them, transferred to a glass bottomed recording chamber mounted on an inverted microscope (Olympus, UK). They were superfused with Hanks solution at 37°C and alternately excited at 340nm and 380nm (PTI Deltascan, NJ, US) via an oil immersion UV objective (x40, NA 1.4). Emitted fluorescence (510nm) was recorded using a photomultiplier tube. Data acquisition, recording, storage and analysis were controlled using PTI FeliX software. Background fluorescence was measured following superfusion with 10mM Mn^2+^ solution and background corrected ratios at 340nm and 380nm (R340/380) used as a measure of intracellular [Ca^2+^]. Drugs were dissolved in dimethyl sulfoxide (DMSO). Superfusion with DMSO alone at the maximum bath concentration used had no effect. Summary data for each protocol represents at least 5 separate cells from at least 3 isolations.

### Electrophysiology

RMECs were seeded onto 35mm culture dishes for 72 hours before performing recordings. These were placed on the stage of an inverted microscope and perfused with the relevant solution at 33°C. Membrane currents were recorded using the whole cell voltage-clamp technique, with an Axopatch 200B amplifier (Axon Instruments, Foster City, CA), and digitized with an analog-to-digital converter (Digidata 1322, Axon Instruments). Data were collected, digitized and analysed using pCLAMP software (version 9, Axon Instruments). Pipette resistances were 3-5MΩ and mean access resistance was 14.9±0.8MΩ. Currents were normalized to cell capacitance (mean cell capacitance was 38.1±1.7pF). The junction potential for the bath and pipette solutions used was measured at 11mV and corrected offline. Drugs were dissolved in DMSO, which had no effect on membrane currents at -80mV and +80mV at the maximum concentration used.

### Solutions and drugs

The composition of the external Hanks’ solution used for Ca^2+^-microfluorimetry experiments was (mM): NaCl, 140; KCl, 6; D-glucose, 5; CaCl_2_, 2; MgCl_2_, 1.3; HEPES, 10; pH set to 7.4 with NaOH. Low Ca^2+^ Hanks’ solution contained no added Ca^2+^ and 1 mM EGTA. External bath solution for electrophysiological recordings was (mM): NaCl, 150; CsCl, 6; CaCl_2_, 1.5; MgCl_2_, 1; D-glucose, 5; HEPES, 10; adjusted to pH 7.4 with Tris. The pipette solution was (mM) Cs-aspartate, 100; CsCl, 20; BAPTA, 10; CaCl_2_, 0.08; Na_2_ATP, 4; MgCl_2_, 1; HEPES, 10; adjusted to pH 7.2 with Tris. Stock solutions of 4α-phorbol 12,13-didecanoate (4α-PDD) and GSK1016790A (Sigma-Aldrich), and HC067047 (Merck Millipore, Watford, UK) were made up in DMSO and dissolved in bath solution to give the required final drug concentration.

### Statistics

Summary data have been presented as mean±standard error of the mean (SEM) throughout. The statistical significance of apparent differences was tested using paired or unpaired t-tests or one-way Analysis of Variance with a Bonferroni post-hoc test corrected for multiple comparisons, with significance set at P<0.05.

## Results

### Molecular and functional expression of TRPV4 in cultured retinal endothelial cells

**Fig 1:**
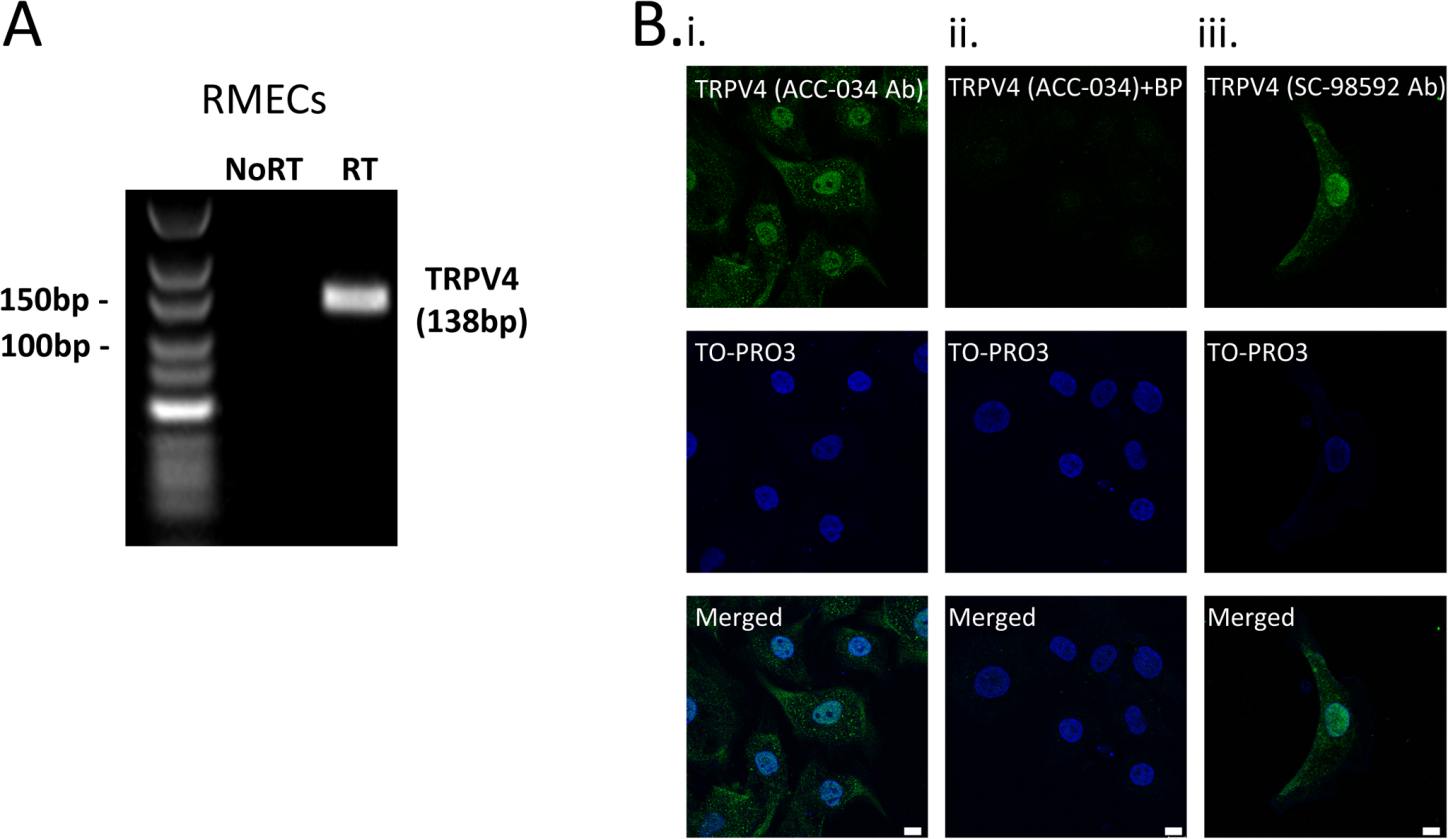
Molecular expression of TRPV4 in retinal microvascular endothelial cells. A. RT-PCR analysis of TRPV4 mRNA expression in cultured bovine retinal microvascular endothelial cells (RMECs). No product was seen when the RT enzyme was omitted (No RT). B(i,ii) Confocal images of RMECs immunostained for TRPV4 (green; ACC-034) and TO-PRO nuclear marker (pseudo-coloured blue) in the absence (i) and presence (ii) of anti-TRPV4 blocking peptide (BP). B (iii) Immunolabelling with a second anti-TRPV4 antibody (SC-98592) revealed a similar pattern of staining in RMECs. Scale bars = 10μm.

We began by confirming the expression of TRPV4 at the mRNA (Fig 1A) and protein (Fig 1B) levels in cultured bovine RMECs. In immunocytochemistry studies, TRPV4 was found to be localized throughout the cells including the nucleus (Fig 1Bi), in a pattern resembling that previously described in cultured human brain capillary endothelial cells[28]. Pre-incubation of the anti-TRPV4 primary antibody with its blocking peptide eliminated nearly all of the immunofluorescence signal indicating that the staining was specific for TRPV4 (Fig 1Bii). An almost identical pattern of TRPV4 staining was observed when RMECs were immunolabelled with a second anti-TRPV4 antibody targeting a different epitope of the channel protein (Fig 1Biii).

**Fig 2:**
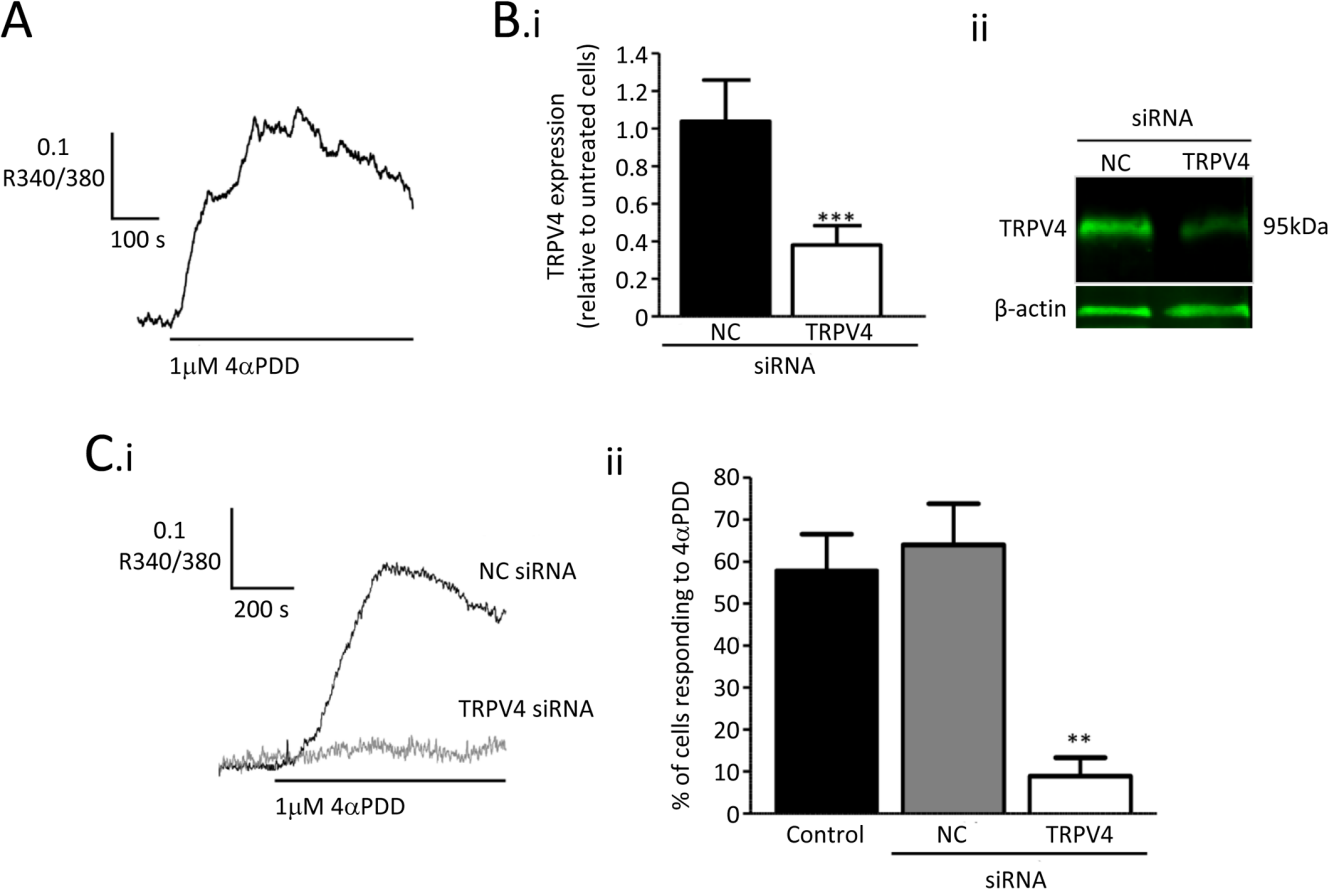
Ca^2+^-responses to the TRPV4 agonist 4αPDD in retinal endothelial cells. A. Fura-2 microfluorimetry record showing a rise in [Ca^**2+**^]_i_ in response to 4αPDD (1μM), as indicated by an increase in the ratio of fluorescence output when alternately excited at 340nm and 380nm (R340/380). B. TRPV4-siRNA inhibited the expression of TRPV4 channels. (i) Summary data from quantitative RT-PCR for TRPV4 mRNA in cells cultured for 72 h after transfection with negative control (NC) or TRPV4 targeted siRNA. Data was normalised to the β-actin control and then expressed as a ratio of the value for untransfected cells in the same experiments (*** P<0.001 v. negative control siRNA). (ii) Representative fluorescent western blots showing reduced TRPV4 expression in RMECs transfected with TRPV4 targeted siRNA when compared with those transfected with negative control siRNA. C. (i) Traces showing [Ca^**2+**^]_i_ changes induced by 4αPDD in transfected RMECs. (ii) Summary data showing the percentage of cells which responded to 4αPDD with a rise in [Ca^**2+**^]_i_ (** P<0.01 v. negative control). A cell response was defined as a peak value for R340/380 during 4αPDD treatment which exceeded the peak value prior to treatment by >2x peak-peak baseline noise.

Experiments were carried out to test TRPV4 channel function in cultured RMECs using fura2 based microfluorimetry (Fig 2A). Superfusion with the TRPV4 agonist 4-αPDD[34,35] increased intracellular [Ca^2+^] ([Ca^2+^]_i_). In 8 similar experiments the mean value of R340/380 was increased by 0.20±0.03, from a baseline of 1.17±0.02 to 1.37±0.02 (P<0.001; paired t-test). The specificity of this response was tested by transfecting RMECs with siRNA targeted against TRPV4. This reduced mRNA by approximately 60% when compared with untransfected cells and with those transfected with a negative control siRNA sequence (Fig 2Bi). Western blots demonstrated that TRPV4 expression was reduced at the protein level (Fig 2Bii; -3.1 fold vs negative control siRNA). The percentage of cells that responded to 4-αPDD was dramatically reduced in cells transfected with TRPV4-siRNA when compared with control cells. No such reduction was seen in RMECs treated with negative control siRNA (Fig 2C).

**Fig 3:**
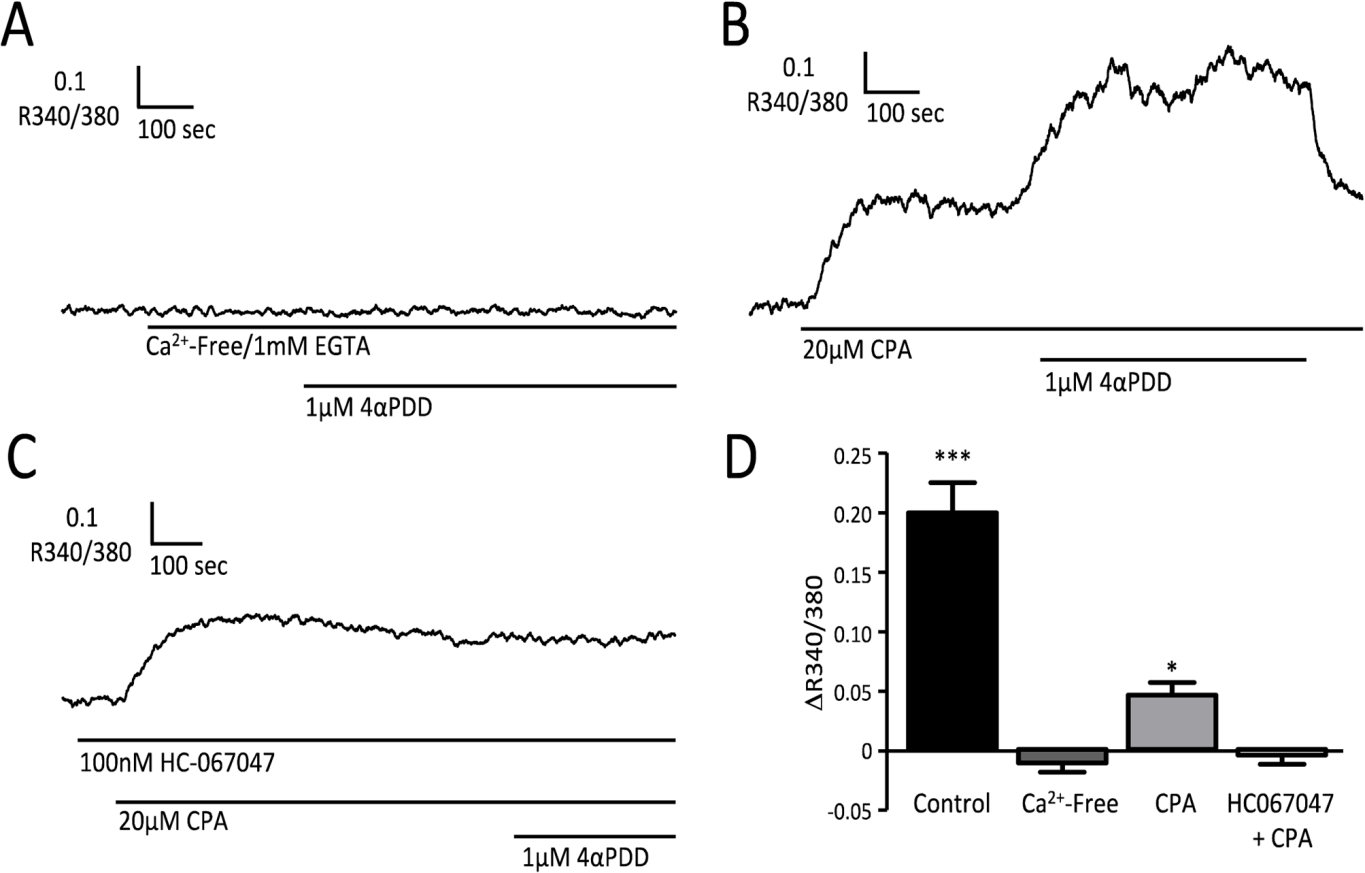
Mechanisms responsible for the Ca^2+^-response to 4αPDD. Microfluorimetry records of [Ca^**2+**^]_i_ changes during application of 4αPDD, (A) in the absence of extracellular Ca^**2+**^ (1 mM EGTA added), (B) in the presence of 20μM CPA, (C) in the presence of 100 nM HC067047 and CPA. (D) Summary data showing the change in R340/380 (ΔR; mean±SEM) in response to 1μM 4αPDD under each of the conditions tested: control (n = 8 cells, replotted from Fig 2A), in a low extracellular [Ca^**2+**^] solution containing 1 mM EGTA (Ca^**2+**^-Free; n = 7 cells), in the presence of 20μM cyclopiazonic acid (CPA; n = 8 cells), and in the presence of both 100nM HC-067047 and CPA (HC+CPA; n = 7 cells). (***P<0.001; *P<0.05 v R340/380 immediately prior to addition of 4αPDD.)

These findings suggest that 4-αPDD raises [Ca^2+^]_i_ in RMECs by a TRPV4-dependent action. The response was completely inhibited by removal of extracellular Ca^2+^, confirming that Ca^2+^-influx through TRPV4 channels in the endothelial cell membrane was necessary (Fig 3A and 3D). However, this does not rule out the possibility that the response might be amplified through Ca^2+^-induced Ca^2+^-release from intracellular stores[36]. To test this, cyclopiazonic acid (CPA; 20μM) was used to empty Ca^2+^-stores. In the presence of CPA, 4-αPDD still produced an increase in [Ca^2+^]_i_, although the amplitude of this response was reduced (Fig 3B and 3D). 4-αPDD increased R340/380 by 0.05±0.01 when added to CPA treated cells (n = 5), as compared with a rise of 0.20±0.03 under control conditions (n = 8, P<0.001, unpaired t-test). CPA alone elevated baseline [Ca^2+^]_i_, presumably through activation of store-operated Ca^2+^-entry (SOCE)[37], with an average increase in R340/380 of 0.05±0.01 (n = 5). This response to CPA was not inhibited by the TRPV4 channel blocker HC067047[38], with a mean rise in R340/380 of 0.11±0.02 (n = 7, NS versus control). The subsequent response to 4-αPDD, however, was completely blocked by HC067047 (Fig 3C and 3D).

**Fig 4:**
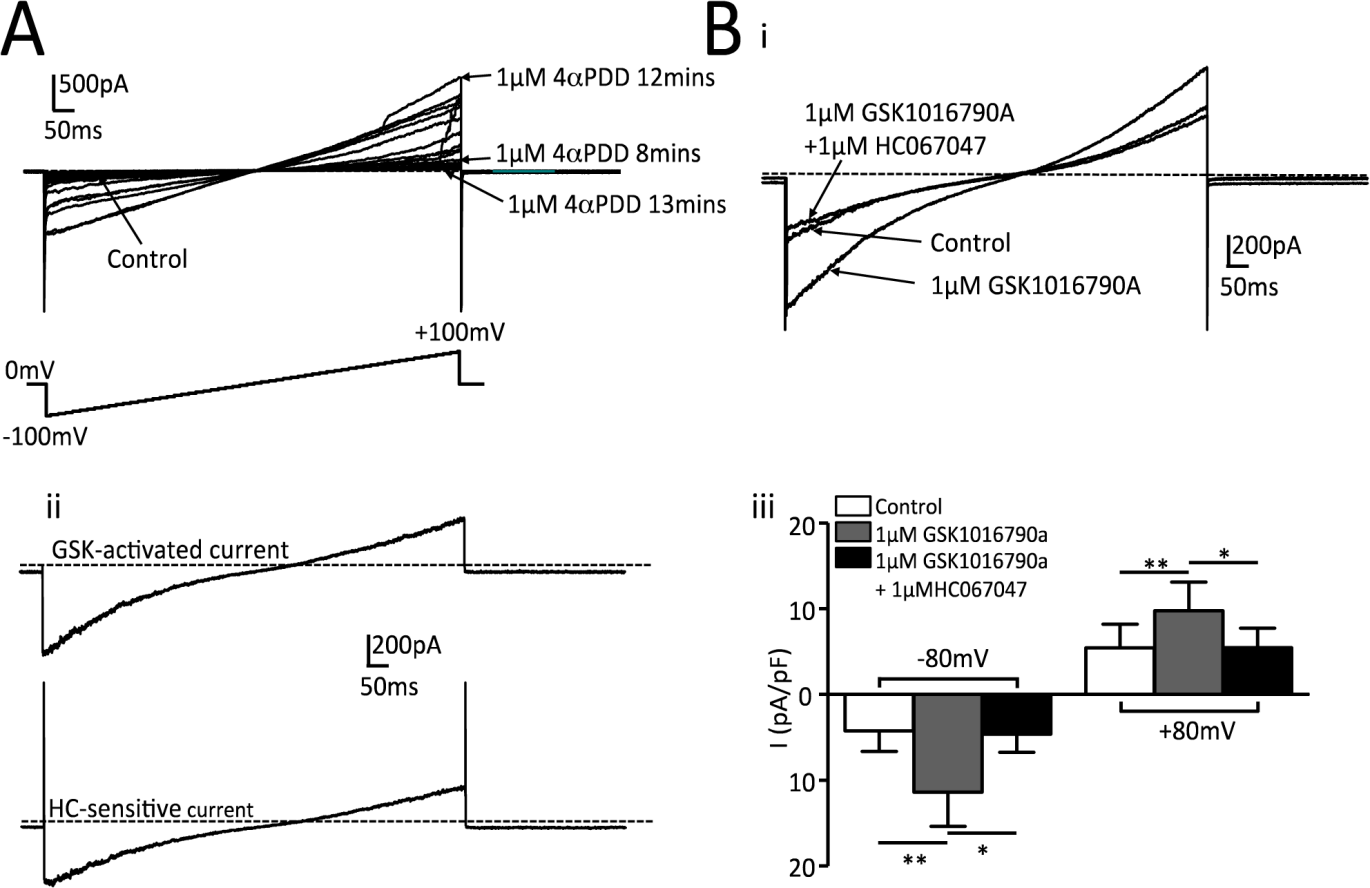
Membrane currents evoked by TRPV4 agonists. A. Whole cell membrane currents recorded during a 1s ramp depolarization from -100mV to +100mV. Application of 1μM 4αPDD activated currents with the expected properties for TRPV4 but these were unstable over time. Control currents were 47pA at -80mV and 40pA at +80mV. B (i) Typical record showing whole cell currents under control conditions, during application of 1 μM GSK1016790A and following subsequent application of 1μM HC067047 in the continued presence of GSK1016790A. ii. GSK1016790A -activated current as defined by the difference between control and GSK1016790A records and HC067047 sensitive current as defined by the difference between GSK1016790A and GSK1016790A + HC067047 records. iii. Summary data for 8 cells from 3 separate cultures for mean current density (±SEM) recorded during steps in membrane potential from 0mV to -80 and +80 mV. (**P<0.01, *P<0.05 for comparisons indicated.)

Functional TRPV4 expression was also assessed using whole-cell recordings of membrane current under voltage clamp conditions (Fig 4). Application of 4-αPDD during ramp depolarizations from -100mV to +100mV elicited whole cell currents that were highly unstable over time (Fig 4A), so further studies were carried out using the TRPV4 agonist GSK1016790A[34]. This activated a current that demonstrated both inward and outward rectification and which was completely inhibited by HC067047 (Fig 4B). The GSK1016790A-difference current had a mean apparent reversal potential of 10.8±1.5mV (Fig 4Bii). Correction for the junction potential (11mV) gave a reversal potential of -0.2mV, close to the predicted value for a non-specific cation current under these recording conditions (-2.1mV).

The amplitudes of the 4-αPDD evoked [Ca^2+^]_i_ rise in the presence of CPA and the GSK1016790A/HC067047 sensitive currents were used as measures of functional expression of TRPV4 in the endothelial cell membrane in subsequent experiments.

### Hyperglycaemia downregulates TRPV4 expression in retinal microvascular endothelial cells

**Fig 5:**
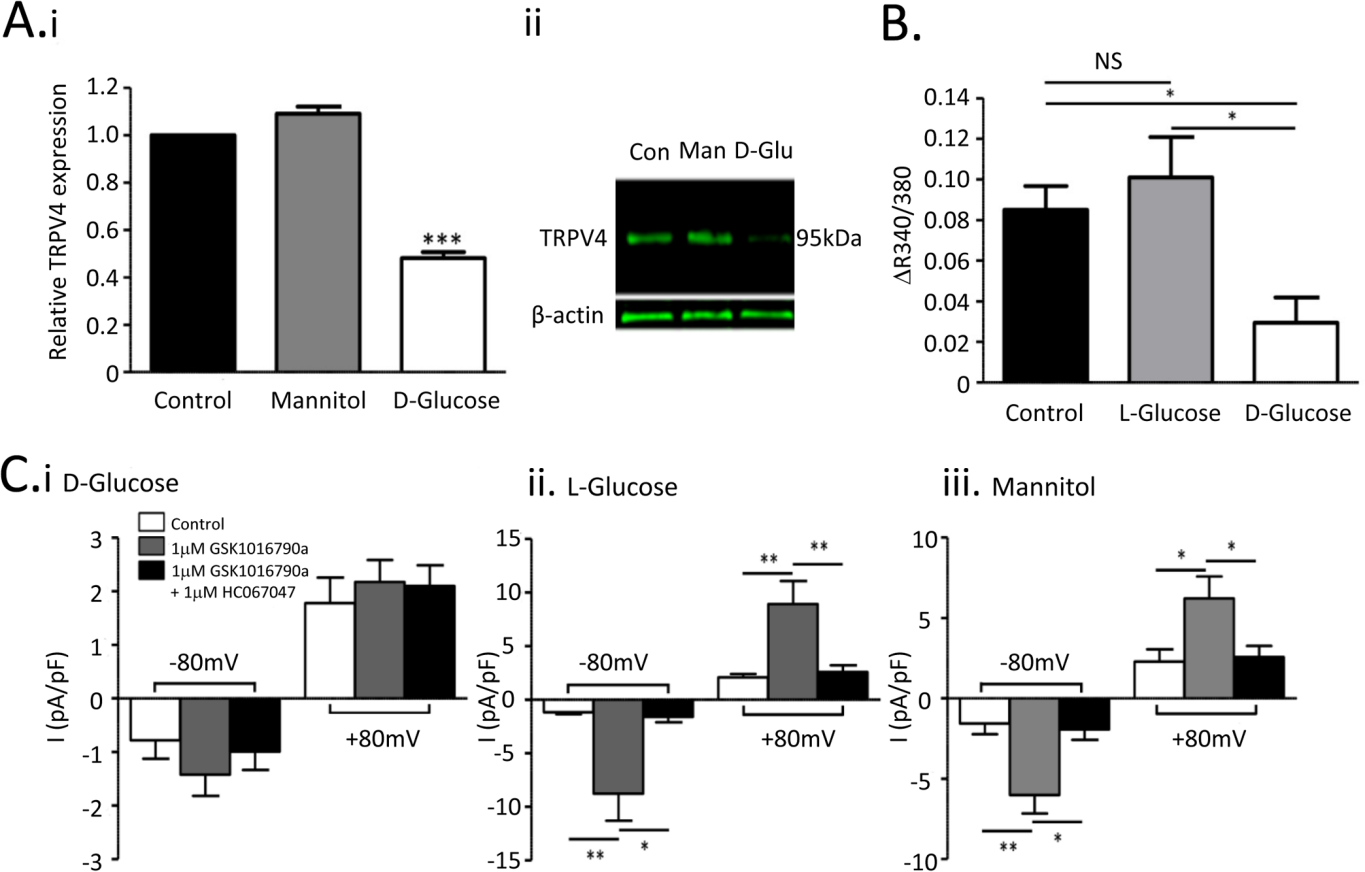
Hyperglycaemia downregulates molecular and functional TRPV expression in retinal microvascular endothelial cells. A. (i) Quantitative real-time PCR for TRPV4 transcript in control RMECs (5mM D-glucose), cells exposed to mannitol (20mM + 5mM D-glucose) and cells exposed to high glucose (25mM D-glucose). (ii) Representative fluorescent western blot showing TRPV4 expression in these same treatment groups. B. Summary data for maximal increases in R340/380 during treatment with 4αPDD. All recordings were made in the presence of 20μM CPA. Mean changes (+SEM) are shown for at least 8 cells from 3 cultures exposed to 5mM D-glucose (control; same data as for CPA in Fig 3D), 20mM L-glucose + 5mM D-glucose (L-glucose) or 25mM D-glucose (D-glucose). C. Summary data for mean cell current density (±SEM) evoked by stepping cell membrane potential from 0mV to -80mV and +80mV (see Fig 4B). Data is shown for control current, current during superfusion with GSK1016790A (1μM) and during superfusion with GSK1016790A (1μM) + HC067047 (1μM). The key for C. i. applies for ii and iii as well. Data is summarised for cells from at least 3 different cultures grown under each condition, i.e., (i) in the presence of 25mM D-glucose, (ii) in the presence of 5mM D-glucose and 20mM L-glucose, and (iii) in the presence of 5mM D-glucose and 20mM mannitol. (**P<0.01; *P<0.05.)

Culturing RMECs for 72 hours in a solution containing 25mM of D-glucose reduced TRPV4 expression at both mRNA (Fig 5Ai) and protein levels (Fig 5Aii; -3.8 fold vs control). Expression was not affected following incubation with an osmotic control containing 5mM D-glucose and 20mM mannitol (Fig 5A; protein levels were 1.3 fold vs control). Hyperglycaemia also inhibited functional TRPV4 responses. Ca^2+^-responses to 4-αPDD were recorded in the presence of CPA, eliminating the contribution from store release (Fig 3), which might also be affected in hyperglycaemia[30]. Under these conditions, the rise in [Ca^2+^]_i_ evoked by 4-αPDD reflected activation of TRPV4 channels functionally expressed in the endothelial cell membrane. Cells grown in 25mM D-glucose produced no significant increase in [Ca^2+^]_i_ during superfusion with 4-αPDD (Fig 5B). Responses recorded from cells incubated with a hyperosmotic control solution containing 20mM L-glucose in addition to 5mM D-glucose were similar to control.

TRPV4 mediated whole-cell currents were used as an alternative measure of TRPV4 activity in the endothelial cell membrane. This was dramatically down-regulated following 72 hours incubation with 25mM D-glucose, with no statistically significant change in current following addition of GSK1016790A at either -80mV or +80mV (Fig 5Ci). In contrast, GSK1016790A activated currents in RMECs incubated for 72 hours with solutions containing 5mM D-glucose supplemented with either 20mM L-glucose (Fig 5Cii) or 20mM mannitol (Fig 5Ciii) were similar to those in control cells. These currents were completely inhibited by HC067047. When corrected for the liquid junction potential, the mean reversal potentials for GSK1016790A-activated currents were -0.1±2.7mV in cells cultured in 20mM mannitol (n = 6) and -3.6±2.3mV in cells cultured with 20mM L-glucose (n = 6). These were similar to those for currents activated in cells cultured under standard conditions (-0.2±1.5mV, n = 8).

### Microvascular expression of TRPV4 is downregulated in diabetic rats

**Fig 6:**
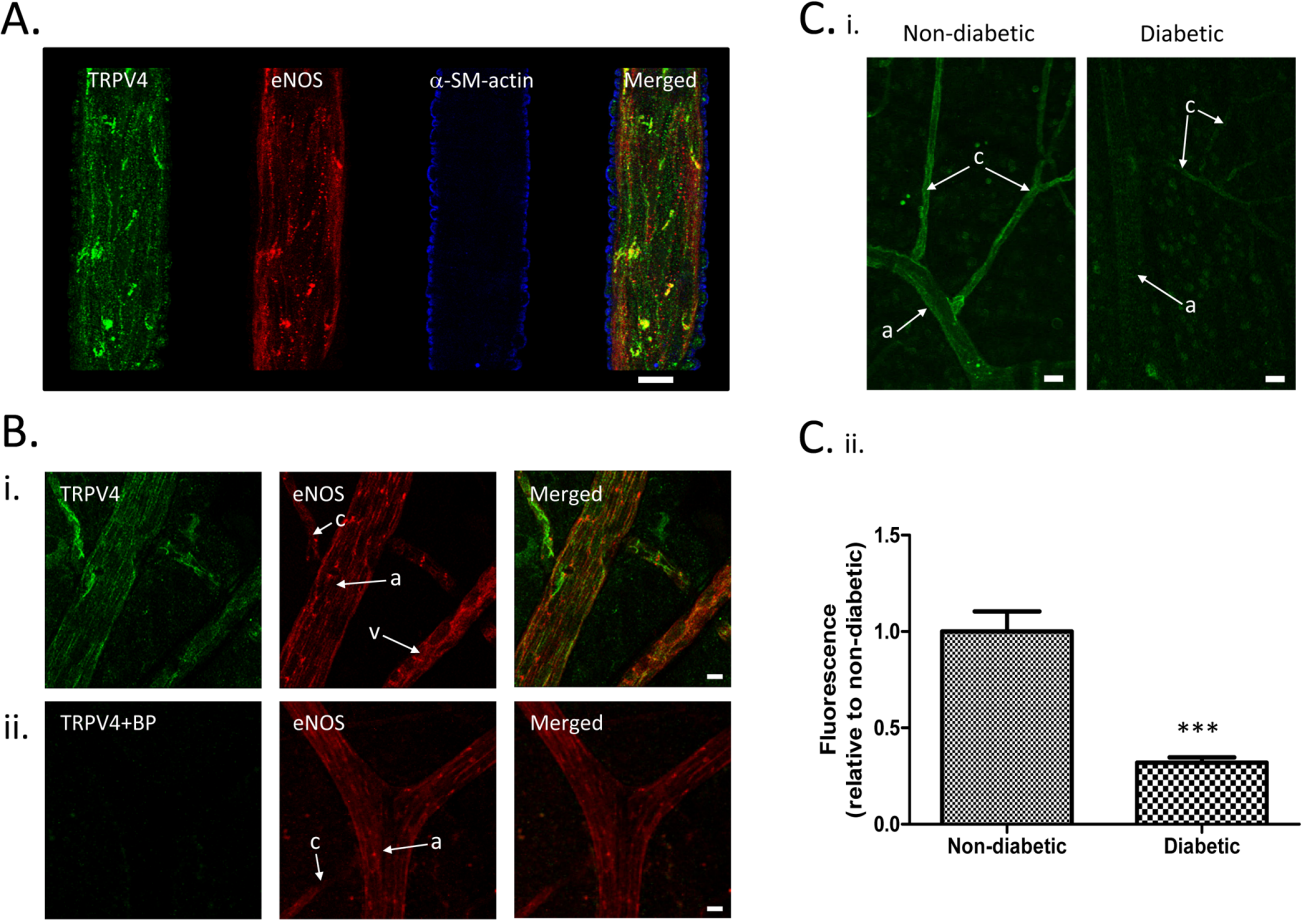
Vascular TRPV4 expression is downregulated in diabetic rats. A. Confocal images of a rat retinal arteriole within a wholemount preparation immunolabelled for TRPV4 (green), eNOS (red channel; endothelial cell marker) and α-smooth muscle actin (pseudo-coloured blue; smooth muscle cell marker). Images have been segmented on the basis of the α-smooth muscle actin expression to specifically isolate the blood vessel staining. Scale bars = 10μm B. Confocal images of rat retinal wholemount preparations immunolabelled for TRPV4 (green) and eNOS (red) in the absence (i) and presence (ii) of anti-TRPV4 blocking peptide (BP). “a” arterioles, “c” capillary and “v” venule. Scale bars = 10μm C(i) Immunohistochemical staining for TRPV4 shows downregulation in both retinal arterioles (“a”) and capillaries (“c”) for diabetic animals. Scale bars = 15μm. (ii) Summary data for TRPV4 immunofluorescence. 4–5 fields of view were averaged for each of 4 animals in 3-month diabetic and age-matched control groups (P<0.001).

A final series of experiments was carried out to investigate the effects of diabetes on retinovascular TRPV4 expression in vivo. We made use of the STZ-rat model, with which we have considerable previous experience[31]. Retinas and retinal arterioles were extracted and processed after 3 months’ diabetes and from age-matched controls. HbA1c values were 6.4±0.3% for the non-diabetic controls and 13.1±0.3% for the diabetic animals (P<0.001, Mann Whitney U-Test). Quantitative RT-PCR demonstrated TRPV4-coding mRNA was reduced by 61.8±2.9% in isolated arterioles when compared with those from control animals (P<0.01; 3 animals per group). Immunohistochemistry of retinal flatmounts from control animals labelled for TRPV4, eNOS and α-smooth muscle actin, demonstrated that TRPV4 is predominantly localized to the endothelium of retinal microvessels, with very little expression in vascular smooth muscle cells (Fig 6A). The endothelial staining of TRPV4 in retinal flatmount preparations appeared specific, since it was not observed when the anti-TRPV4 primary antibody was pre-incubated with its blocking peptide (Fig 6B). Consistent with our quantitative RT-PCR data, semi-quantitative analysis of immunolabelled flatmount preparations indicated that TRPV4 expression was downregulated at the protein level in retinal microvessels, with decreased endothelial staining in arterioles and capillaries (Fig 6C). TRPV4 Immunofluorescence was decreased by 68.1±2.8% (P<0.001; n = 4 retinal preparations for both diabetic and non-diabetic animals).

## Discussion

In this study we have demonstrated that TRPV4 channels are expressed at the molecular and functional level in cultured retinal endothelial cells (Figs 1–4). Two independent techniques for the assessment of TRPV4 channel function were validated. Endothelial Ca^2+^-responses to 4-αPDD were almost completely abolished following siRNA knock-down of TRPV4, providing strong evidence of agonist specificity (Fig 2). These responses were also suppressed by removal of extracellular Ca^2+^, indicating that Ca^2+^-entry through channels in the cell membrane was a necessary step (Fig 3). The use of CPA to empty the intracellular stores dramatically reduced but did not abolish the rise in Ca^2+^, indicating that the TRPV4 Ca^2+^-signal was substantially dependent on store release. Although this protocol could not distinguish between direct activation of intracellular TRPV4 channels by 4-αPDD and signal amplification through Ca^2+^-induced Ca^2+^-release[36], it did allow the activity of functional TRPV4 channels in the endothelial cell membrane to be evaluated. Under these conditions, the response to 4-αPDD was completely inhibited by the TRPV4 antagonist HC067047, consistent with the presumed target specificity of both agonist and inhibitor (Fig 3C and 3D). HC067047 had no effect on the amplitude of the CPA-induced Ca^2+^-rise in these cells, suggesting that TRPV4 does not play a significant role in store-operated Ca^2+^-entry. Previous studies suggest that heteromeric TRPV4-TRPC1 channels can play a role in store operated Ca^2+^ entry in cerebral microvascular endothelium[39]. The absence of any effect by HC067047 on CPA-responses in RMECs may reflect either a minimal contribution from TRPV4 containing channels or limited block of heteromeric channels by this drug.

Functional TRPV4 expression in the cell membrane was also assessed directly by recording membrane ion currents (Fig 4). Consistent with the Ca^2+^-signalling data above, 4-αPDD-activated currents were observed, but these were highly unstable over time. GSK1016790A, another potent and selective TRPV4 activator[34,40], stimulated a current that was completely inhibited by HC067047 and reversed close to the expected reversal potential for a non-specific cation current under the experimental conditions used. This had characteristics similar to TRPV4 currents seen in expression systems and wild type vascular endothelium, with both inward and outward rectification[22,28,41,42]. The GSK1016790A difference current allowed TRPV4 channel activity to be isolated from that of other endothelial ion channels[43]. TRPV4 has been shown to be significantly activated at temperatures >25°C in mouse aortic endothelial cells[22,42]. Since the experiments described here were carried out at 33–37°C, it might be expected that most TRPV4 channels might have already been ‘constitutively’ open under basal conditions. However, a number of our observations suggest that this was not the case. Both 4-αPDD and GSK1016790A stimulated cell responses, suggesting activation of additional TRPV4 channels. This is consistent with findings in cultured human coronary endothelial cells in which 4-αPDD evoked Ca^2+^-responses at 37°C[16] and with observations from TRPV4 expressing HEK293 cells, in which 4-αPDD stimulated a five-fold greater current than heating to 38°C[42]. HC067047 had no effect on basal [Ca^2+^]_i_ (Fig 3C) and only reduced the GSK1016790A activated currents back to, but not below, control levels (Fig 4B). Limited basal activation of TRPV4 may also help explain why removal of external Ca^2+^ alone had little or no effect on [Ca^2+^]_i_ (Fig 3A and 3D). Most importantly, these findings support the proposal that TRPV4 may play a role in endothelial signaling at physiological temperatures.

Endothelial expression of TRPV4 mRNA and protein were downregulated in RMECs grown under high glucose conditions (Fig 5) and in retinal vessels from diabetic rats (Fig 6). This was correlated with a dramatic reduction in TRPV4 function as assessed using [Ca^2+^]_i_-responses to 4-αPDD and GSK1016790A/HC067047 sensitive membrane currents (Fig 5). Hyperosmotic control solutions had no effect on TRPV4 expression or function, an important point, given that TRPV4 channels are sensitive to osmolarity in a variety of cell types, including vascular endothelium[41,44]. While direct inhibition of channels under high glucose conditions cannot be ruled out, it seems likely that the loss of TRPV4 function resulted from reduced channel expression. Moreover, the loss of TRPV4 expression observed in the retinal vasculature of STZ-rats of 3-months disease duration cannot be simply attributed to endothelial cell dropout, since it is well established that there is no significant retinal endothelial cell loss in this model at this time point[45–48]. Our findings are consistent with a recent study on mesenteric arterial endothelium reporting downregulation of TRPV4 expression in STZ diabetic rats [20]. The current findings suggest that diabetes reduced both TRPV4 expression and function in the retinal microcirculation, an important site of microvascular pathology[49].

Given their key role in endothelial signalling, downregulation of TRPV4 function might be expected to have significant vascular consequences. TRPV4 channels have been implicated in endothelium dependent vasodilatory responses to a wide range of stimuli, including increased flow and shear stress[16–19], agonist activation [20,21] and reduced intravascular pressure [15]. Intravenous administration of TRPV4 activators results in hypotension and can lead to circulatory collapse, which is at least partly explained by increased capillary permeability and fluid loss, particularly in the lungs[50–53]. Experimental deletion of the TRPV4 gene in mice reduces the hypotensive response to acetylcholine [54] and increases the hypertensive effects of inhibition of nitric oxide synthase[23]. Interpretation of these observations, however, is complicated by the fact that TRPV4 is expressed on vascular smooth muscle in some vessels (although in the present study only limited expression of these channels was observed in vascular smooth cells of the retinal vasculature; Fig 6A). Activation of these channels may affect vascular tone in an endothelium independent manner [23,25]. At the whole body level, the picture is even more complex, since TRPV4 is also expressed in non-vascular tissue. For example, regulation of fluid and osmotic balance is impaired in knockout mice secondary to loss of osmotically activated TRPV4 channels in the CNS [23,55]. This may help explain why these animals do not show a hypertensive phenotype, as might be expected given TRPV4’s vasodilatory role. This underlines the need for a tissue specific knock out model if the normal role of endothelial TRPV4 is to be dissected out.

In summary, we have provided direct evidence of functional TRPV4 expression in the cell membrane of the retinal microvascular endothelium and demonstrated that this expression is downregulated both by hyperglycaemic culture conditions in vitro and by diabetes in vivo. Given that TRPV4 signalling has been demonstrated to play a role in several types of endothelium-dependent vasodilatation, it is tempting to speculate that loss of TRPV4 function may contribute to endothelial dysfunction in diabetes. The contribution of TRPV4 channel downregulation to the loss of endothelium-dependent vasodilatation and the pathogenesis of diabetic retinopathy now warrants further investigation.
